# Development and Testing of a 5G Multichannel Intelligent Seismograph Based on Raspberry Pi

**DOI:** 10.3390/s22114193

**Published:** 2022-05-31

**Authors:** Igbinigie Philip Idehen, Qingyu You, Xiqiang Xu, Shaoqing Li, Yan Zhang, Yaoxing Hu, Yuan Wang

**Affiliations:** 1 Key Laboratory of Petroleum Resources Research, Institute of Geology and Geophysics, Chinese Academy of Sciences, Beijing 100029, China; qyyou0880@mail.iggcas.ac.cn (Q.Y.); xuxiqiang@mail.iggcas.ac.cn (X.X.); lisq@mail.iggcas.ac.cn (S.L.); zhangyan@mail.iggcas.ac.cn (Y.Z.); yxhu@mail.iggcas.ac.cn (Y.H.); ywang@mail.iggcas.ac.cn (Y.W.); 2 Department of Physics and Physics Electronics, Anchor University, Ayobo P.M.B 00001, Lagos 100278, Nigeria; 3 Department of Geology, Obafemi Awolowo University, P.M.B 13, Ile-Ife 220282, Nigeria

**Keywords:** Raspberry Pi, multichannel seismograph, ambient noise tomography, IoT, real-time data transfer, 5G module, machine learning

## Abstract

A seismograph was designed based on Raspberry Pi. Although comprising 8 channels, the seismograph can be expanded to 16, 24, or 32 channels by using a USB interfacing with a microcontroller. In addition, by clustering more than one Raspberry Pi, the number of possible channels can be extended beyond 32. In this study, we also explored the computational intelligence of Raspberry Pi for running real-time systems and multithreaded algorithms to process raw seismic data. Also integrated into the seismograph is a Huawei MH5000-31 5G module, which provided high-speed internet real-time operations. Other hardware peripherals included a 24 bit ADS1251 analog-to-digital converter (ADC) and a STM32F407 microcontroller. Real-time data were acquired in the field for ambient noise tomography. An analysis tool called spatial autocorrelation (SPAC) was used to analyze the data, followed by inversion, which revealed the subsurface velocity of the site location. The proposed seismograph is prospective for small, medium, or commercial data acquisition. In accordance with the processing power and stability of Raspberry Pi, which were confirmed in this study, the proposed seismograph is also recommended as a template for developing high-performance computing applications, such as artificial intelligence (AI) in seismology and other related disciplines.

## 1. Introduction

Seismology dates back more than 4000 years, when men became curious about the reasons for earthquakes, volcanoes, tremors, and other related environmental activities [1,2]. These curiosities opened doors for the development of early seismic data acquisition systems [3]. From the mid-twentieth century to date, there have been significant advancements in the fields of information and communications technology, which have enabled the development of more advanced seismic data acquisition systems, including seismographs and geophysical sensors, which are now available to study the earth’s interior and to conduct other geophysical explorations. The aforementioned technology has also yielded other benefits, such as the development and availability of modeling, processing, and interpretation applications, as well as software in the field of geosciences.

As is commonly understood, a seismograph collects, transmits, stores, processes, and displays seismic data either from real or virtual sources. Any shortcomings or defects in the development process of seismographs will no doubt render the seismic data useless, and time and resources would, as a result, be lost. If problems in the design of a seismograph are not resolved, data processing and interpretation would be handicapped by extension. If such an error occurs in regional seismology, the loss of time, resources, and data could be monumental and difficult to recover from. To avoid these cascading effects, geophysical instruments must be continually developed through sustained research efforts.

A number of research studies on seismograph design, construction, and testing have been carried out to date [4,5,6,7,8,9,10]. Notwithstanding the integration of state-of-the-art IoT technology and high-speed, real-time systems, 5G technology has not been given sufficient attention, especially in geophysical instrument development. Additionally, the processing ability, stability, and other related potentials of Raspberry Pi with respect to running 5G high-speed multichannel seismographs, multithreaded algorithms, and computer-intensive embedded applications, represent not just a research contribution to the field of geosciences but also benefit hobbyists, artificial intelligence (AI), and other related fields.

According to past and recent research studies, Raspberry Pi has potential beyond used as a microcontroller; it is a full-fledged computer. As long as there is a compiler or interpreter, application software such as Python, C++, PHP, or Java can run on Raspberry Pi. Its latest version released in 2019, Raspberry Pi 4, which is more IoT-friendly than its predecessors, was used for this study. With many merits, its latest version is well-known to have three RAM variants, 1 GHz, 2 GHz, and 4 GHz, making it suitable for developing memory-intensive algorithms. In addition to its portability and mobility, Raspberry Pi is easily scalable, opening the possibility of user-friendly channel expansion for multichannel seismographs. Additionally, by clustering Raspberry Pi through parallel computing technology, the number of possible channels can be increased by at least 10-fold, enabling flexible and robust nodes [11,12,13]. Its quad-core 64 bit processor has enabled a number of contemporary studies, especially with respect to the development and implementation of smart and intelligent monitoring systems, such as that exemplified in this study [14,15,16,17,18].

The integration of 5G modules into Raspberry; the stability of Raspberry Pi in high-speed, real-time systems; its computational intelligence with respect to multithreaded algorithms; its scalability for small-to-commercial seismic data acquisition; and the prospect of developing seismological predictions for modelling purposes makes it a worthy research object.

## 2. System Description

A multichannel seismograph comprising eight channels with IoT features was designed and tested. A Raspberry Pi, a Huawei MH5000-31 5G module, an STM32F407 microcontroller, and a ADS1251 delta-sigma ADC from Texas Instruments were the main devices configured and interfaced together. High-speed internet needed to drive the IoT was provided by the 5G module. The STM32F407 microcontroller is a low-power 32 bit Cortex-M4 processor with FPU instruction sets and core peripherals. It supports processor clock of up to 168 MHz, 512 KB flash memory, and 196 KB of SRAM. The ADS1251 ADC has 24 bit resolution with a programmable oversampling rate of up to 20 kHz and a wide dynamic range of 144 dB [19]. The two-wire serial peripheral interface (SPI) of each of the eight ADCs makes programming with the microcontroller uncomplicated.

**Figure 1:**
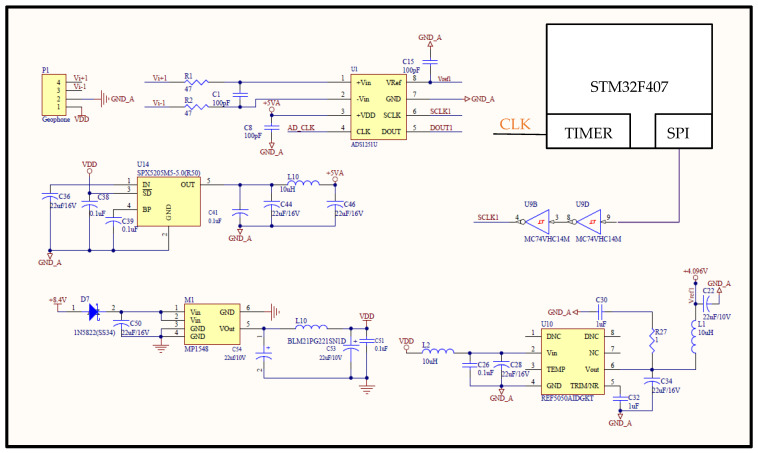
Schematic of the hardware peripherals used for the proposed seismograph. Only one of the ADCs is shown.

A description of the first phase of the seismograph system hardware is presented in Figure 1. Starting with the power supply, two rechargeable 8.4 V lithium batteries were connected in parallel. The resulting 8.4 V output was passed through a schlocky diode, then to the MPI584 DC–DC converter, stepping it down to 5 V VDD [20]. Ready-made geophones with inbuilt signal conditioning circuits were connected to the differential input terminals of the ADS1251 ADCs through a 47 ohm resistor. A 100 pF bypass capacitor was connected in parallel to both resistors of the ADC and placed as close as possible to its differential inputs to minimize noise and stabilize the analog from the geophone. Also connected to the ADS1251 was a low-noise SPX5205 positive voltage regulator with an output tolerance of less than one percent. The voltage regulator provided a stable 5 V power supply for the ADCs. Another chip, a Texas Instruments REF5040 high-precision 4.096 V reference, was connected to the analog voltage reference pin of all the ADS1251 ADCs. This voltage reference is critical to maintain an accurate ADC output. The voltage reference was connected across filter capacitors and inductors so as not to degrade its outputs. Each ADC had two Schmitt inverter inputs pins, a serial clock (SCLK), and a system clock (CLK). A high-immunity-to-noise and stable-output MC74VHC14 Schmitt inverter was connected to both of these pins. One of the general-purpose timers of the microcontroller was programmed in pulse width modulation (PWM) mode with a frequency of 0.0768 MHz after dividing by a prescaler provided the needed CLK at 50% duty cycle. According to the ADS1251 specifications, this will produce a data output rate of 200 Hz.

**Figure 2:**
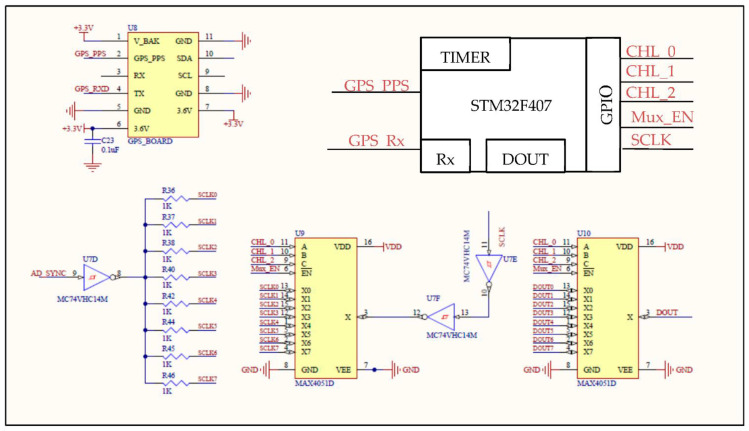
Schematic of the additional hardware peripherals used for the proposed seismograph.

The hardware description of the seismograph is further extended in Figure 2. Besides clocking out data, the SCLK is used to put multiple ADCs in synchronization mode. Synchronization is crucial, as an error in synchronization will cause an error during simultaneous sampling of the analog input signals. During synchronization, the data-ready pins (DRDYs) of the ADCs were in phase. After synchronization, the SCLK output was fed into the Schmitt inverter inputs twice to reinforce its output. Two MAX4051D analog multiplexers were used to read outputs of the two SPI pins, SCLK, and the serial data out (DOUT). The channel-select pins (CHL_0–CHL_2) and the enable pin ((Mux_EN)) of the multiplexer were all connected to the GPIO pins of the microcontroller with correct configuration in accordance with datasheet specifications and reference manuals. A Ublox NEO-7M, a global navigation satellite system (GNSS) receiver was used to read GPS time via the universal asynchronous receiver–transmitter (UART) communication protocol [21]. One of the timers of the microcontroller configured in input capture mode with a frequency of 1 Hz was enabled to read the GPS receiver pulse per second (PPS) output. With the help of PPS, the microcontroller time was synchronized to that of the GPS, which is exceptionally accurate. Additionally, the digital and analog ground of all the peripherals on the printed circuit board (PCB) were routed separately and connected to a common ground through a zero-ohm resistor, in order to reduce noise on the PCB. A TP5100 chip [22] and a step-down lithium battery charger soldered in dual mode were included on the PCB for recharging purposes. 

The firmware for the microcontroller was written in embedded C language using the Keil µVision5 IDE. Starting with synchronization, the SCLK is low by default. However, holding the SCLK at high for at least 1536CLK but not more than 7680CLK will sync all the ADCs. A CLK frequency of 0.0768 MHz resulted in a delay range of 2.4868–12.4343 ms and synched all the ADCs. The AD_SYNC GPIO pin controlled this delay using one of the timers of the STM32F407 configured with interrupt. Both DRDY and DOUT pins are separated by a 10 ohm resistor with the DRDY connected to one of the external interrupt pins of the microcontroller. As the name suggests, the DRDY is used to indicate when new data are available to be read from the ADS12251 data register. Afterward, the data are clocked-out to the DOUT pin by SCLK. Digital filtering of the raw ADC data was performed using a finite impulse response low-pass filter prior to serial data transmission to the Raspberry Pi. With the help of the USB communication device class (USB CDC) protocol, read data from the ADC with periodic timestamps was sent to the Raspberry Pi for possible simultaneous data transfer to a web server, Alibaba IoT cloud, and the Raspberry Pi SD card.

**Figure 3:**
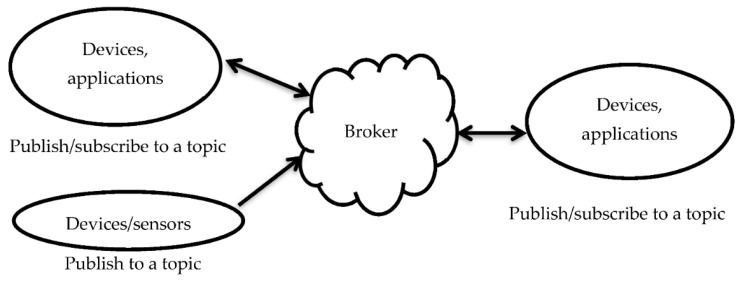
Architecture of the MQTT protocol. Many devices that can subscribe can also publish, but the reverse is not always the case.

A Huawei MH5000-31 5G module was used that supports Windows or Linux environments, hot plugs, and multiple modes (2G/3G/4G/5G networks). Specifically, it was used for real-time data plotting on a web browser and real-time data transfer to Alibaba cloud broker using the message queuing telemetry transport (MQTT) protocol. The MQTT protocol used for real-time data transfer to the Alibaba cloud broker is a low-bandwidth protocol based on data subscription and publication from clients, with a broker serving as the middle man between them (Figure 3). Access to the MQTT broker from which subscribed data can be written to a file in real time was programmed based on the STOMP Python library.

## 3. System Testing and Field Setup

**Figure 4:**
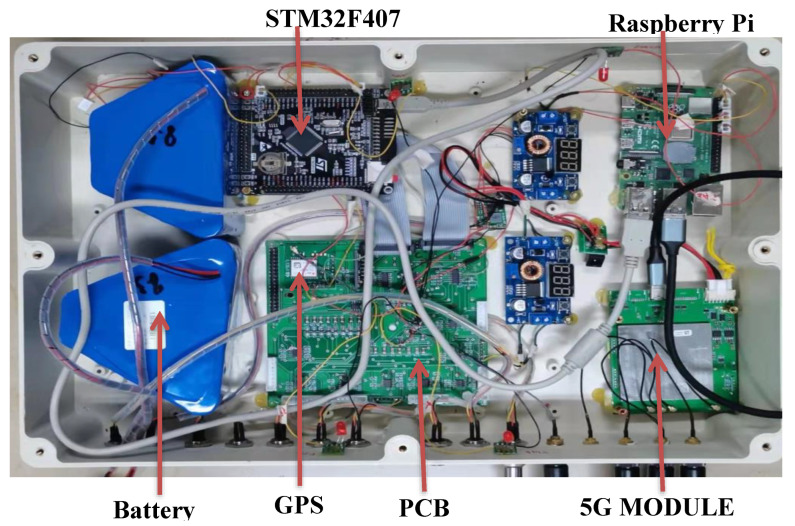
Components of the seismograph assembled in a box.

**Figure 5:**
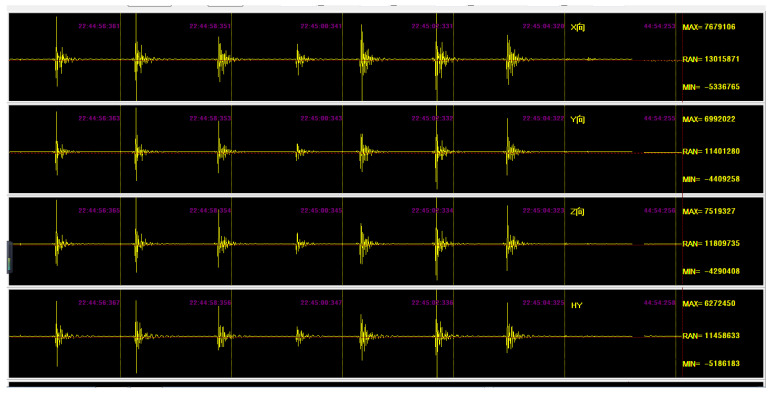
Preliminary laboratory testing of the seismograph for four channels only. The seismic source was banging.

The PCB, the STM32F407 development board, the Raspberry Pi, and the 5G module were assembled in a box (Figure 4). Before field testing of the multichannel seismograph, a four-channel application for live streaming of seismic data waveforms was designed using Delphi Pascal software and used to display the ADC output. The maximum, minimum, and range of ADC values were displayed in a golden-colored display on the right side of each row (Figure 5).

**Figure 6:**
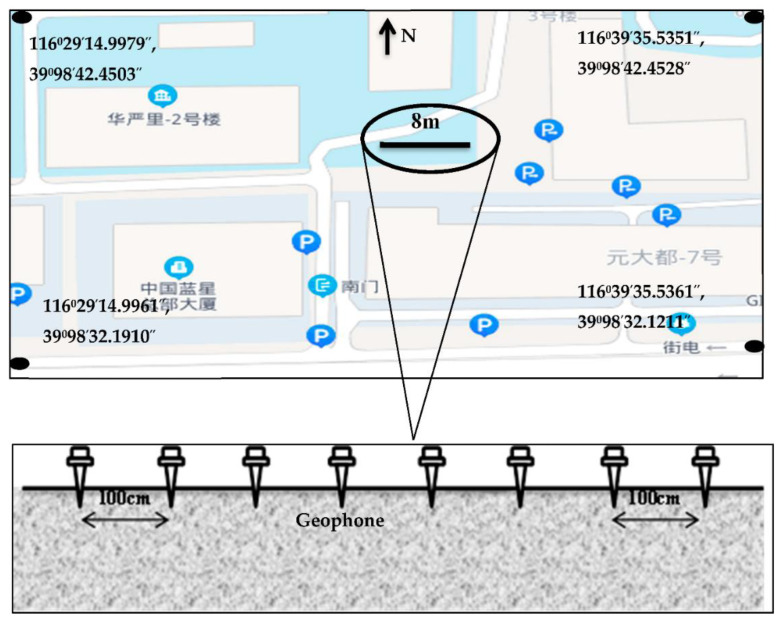
Field testing of the proposed multichannel seismograph. A single traverse of 8 m spread was used for seismic wave tomography. Each geophone was planted vertically with an offset of 1 m from its neighbors.

The premises of the Institute of Geology and Geophysics, Chinese Academy of Sciences, Beijing, China were used for the field test (Figure 6). The testing site is close to metro tunnels and towering buildings. The environment also has regular urban metro traffic, vehicular movements, and other related anthropogenic activities. The seismic waves from background noise were recorded for all eight channels with a total traverse length of 8 m using vertically planted geophones spaced by 1 m.

**Figure 7:**
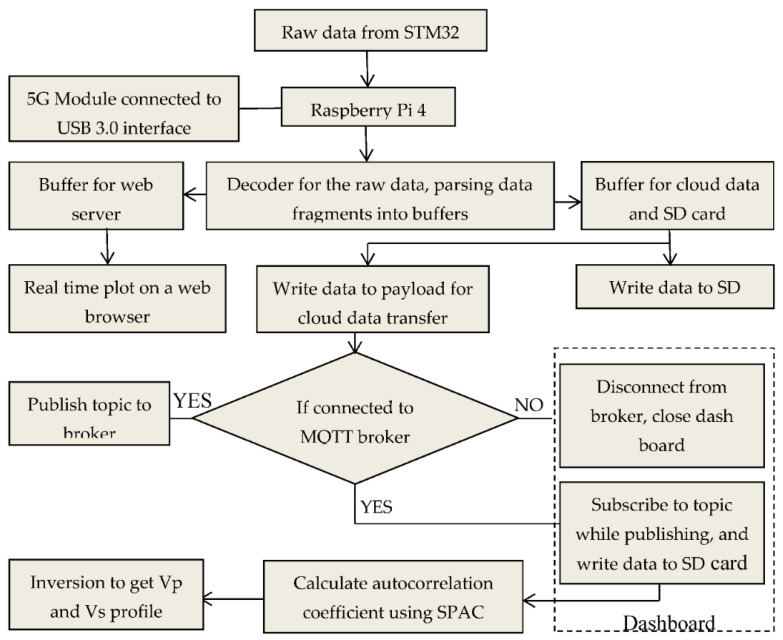
Flow chart of the algorithms and real-time system using Raspberry Pi.

A flow chart of the intelligent seismograph is shown in Figure 7. The raw seismic data from the multichannel seismograph are transmitted from the microcontroller to the Raspberry Pi via USB CDC. The data are received in byte strings and are preprocessed and distributed to two separate buffers: one for the web server and the other for simultaneous data transfer to the SD card and the Alibaba cloud broker. By using the flask library, real-time data for all eight channels are plotted on a web broker. This output helps to first ascertain the fidelity of the seismic data, preventing junk data from being sent to the cloud broker or SD card. Relying only on real-time data transfer to the cloud broker could result in failure due to a poor network connection. Therefore, the alternative of directly saving to the SD card could salvage the situation from total loss and waste.

## 4. Result and Interpretations

**Figure 8:**
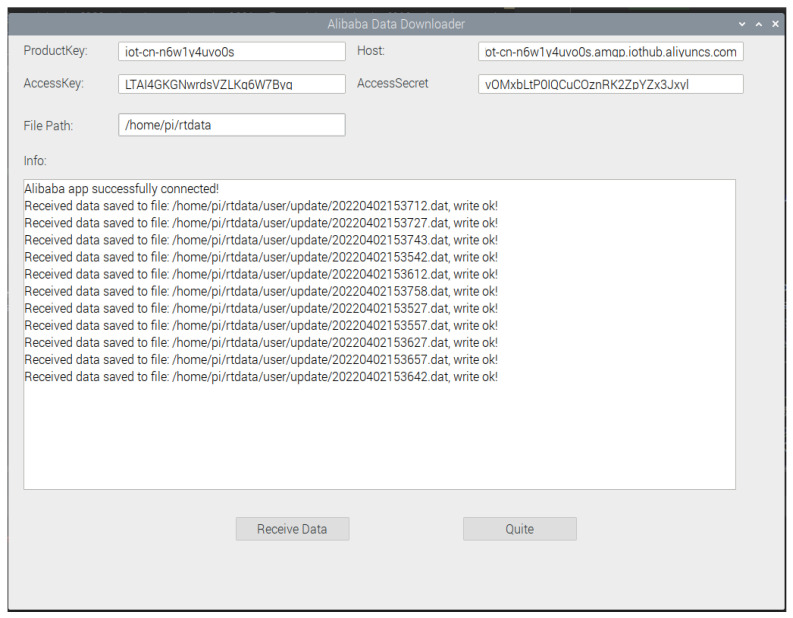
Screenshot of the dashboard designed using the wxPython library. Subscribed data were downloaded in real time using this application by clicking on the “Receive Data” button. The “Quite” button terminates the data transmission.

**Figure 9:**
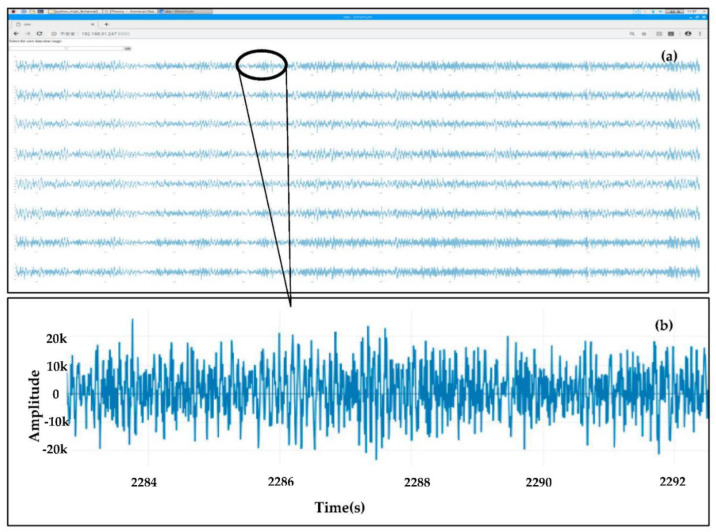
Screenshots from live streams of the seismic waveforms for the eight-channel seismograph running on the web browser of the Raspberry Pi. (**a**) Generated from ambient noise. (**b**) A zoomed-in section of channel 1.

**Figure 10:**
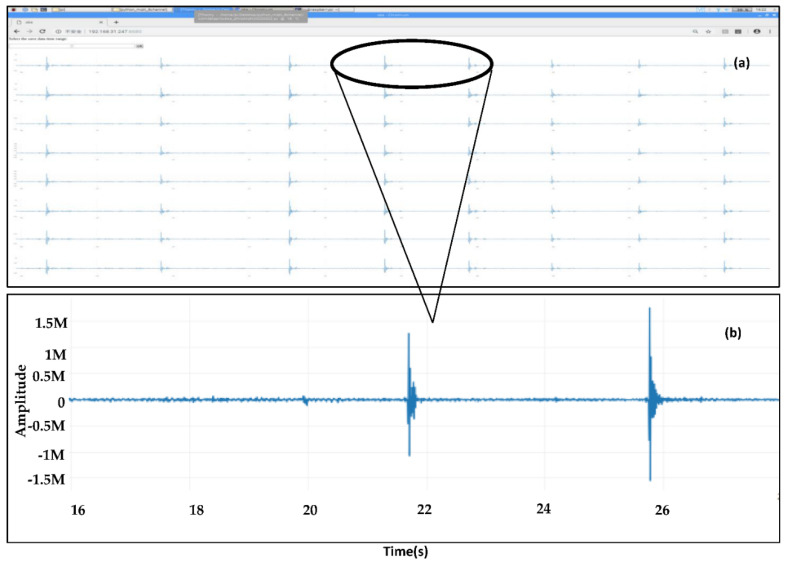
Screenshots from live streams of the seismic waveforms for the eight-channel seismograph running on the web browser of the Raspberry Pi. (**a**) Generated by banging. (**b**) A zoomed-in section of channel 1.

A functional dashboard based on a wxPython module controlled the data subscription from the cloud broker (Figure 8). Remote viewing software, TeamViewer, provided a remote-control window. The received seismic waveforms were displayed in the time domain of a web browser of the Raspberry Pi, as shown in Figure 9 and Figure 10, and the seismic waveforms appeared as expected.

**Figure 11:**
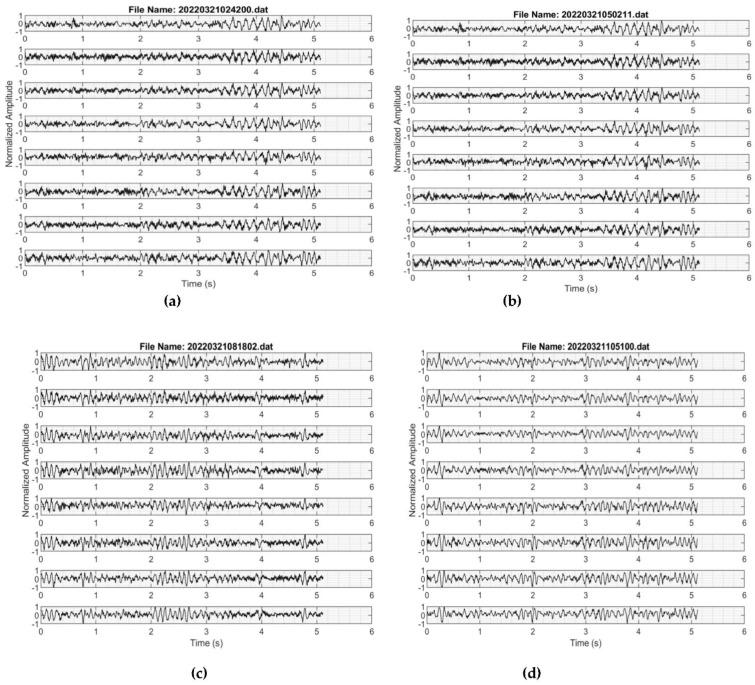
Real-time seismic waveforms (during different time windows) for the proposed eight-channel seismograph using the previously described field setup. (**a**) At about 20220321024200 + 8 h UTC time (Beijing, China). (**b**) At about 20220321050211 + 8 h UTC time (Beijing, China). (**c**) At about 20220321081802 + 8 h UTC time (Beijing, China). (**d**) At about 20220321105100 + 8 UTC time (Beijing, China).

**Figure 12:**
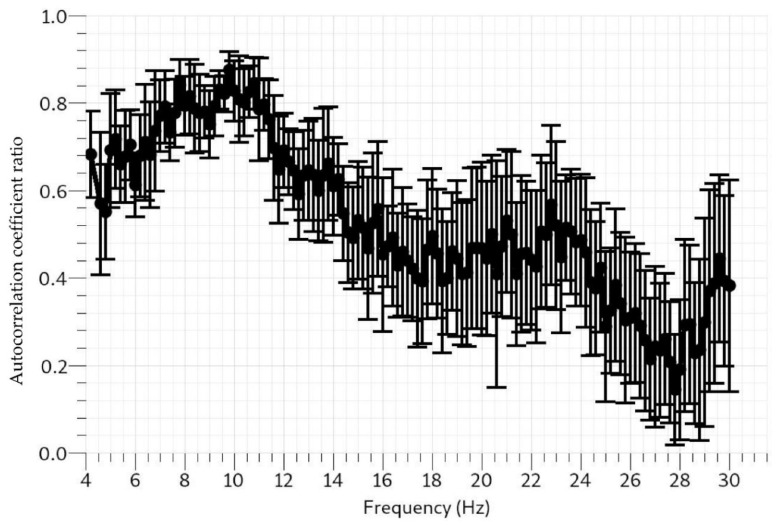
Spatial autocorrelation coefficient ratio vs. frequency plot shows the SPAC curve.

**Figure 13:**
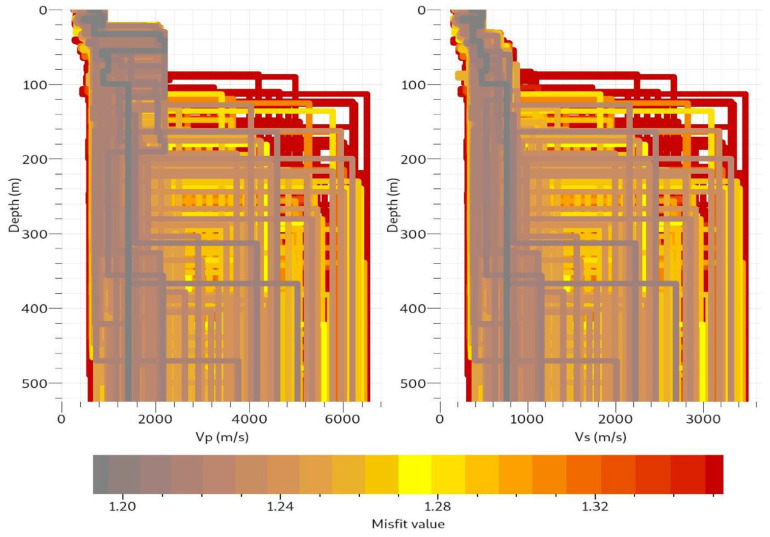
Velocity modeled for both Vp and Vs as obtained from the SPAC curve. Multiple layers were revealed, with a maximum depth of about 500 m.

The seismic waveforms of the real-time data transmitted to the cloud broker are presented in Figure 11a–d. The consistency and negligible line drift are obvious from different time windows. Vibrations from urban metros running across tunnels and the many anthropogenic activities in the test location, Beijing, the Chinese capital, could be responsible for the high-amplitude signals recorded by the vertically planted geophones [23,24,25]. Using Figure 11a as an example, (also applicable to Figure 11b–d) the file name, 20220321024200.dat, gives the UTC real-time metric at which the data were recorded. 2022 represents the year, 03 is the month, 21 is the day, 02 is the hour, 42 represents minutes, and 00 stands for seconds. Although each file comprises 1024 data points at a 200 Hz sampling rate, the data were transmitted with relevant information from the timestamps and data headers. The real-time ambient noise data from the cloud broker were processed using the modified spatial autocorrelation (SPAC) method. SPAC has been used in the past to analyze ambient noise data from which inversion can be carried out [26]. The SPAC toolbox is included in Geopsy open-source software, which is used to obtain the spatial autocorrelation coefficients at different frequencies, as shown in Figure 12. Additionally, the p-wave (Vp) and s-wave (Vs) velocity models of the subsurface obtained by inversion are displayed in Figure 13.

Raspberry Pi has good scalability, and it increases with the number of nodes. Similarly, due to its clustering ability, embedded parallelism, and versatile technology for channel expansions, this seismograph is recommended for portable, mobile, and cost effective commercial data acquisition requiring low power consumption [27,28,29].

## 5. Conclusions

The findings of this study show the processing intelligence of Raspberry Pi to process real-time data transfer at high speed using interfaced 5G technology, run a web server application using real time-seismic data, and execute cloud computing algorithms. This was further confirmed by a field test and processed results. The result obtained from the real-time data were reliable and found to be stable over different time windows. The proposed seismograph has good scalability, as the number of channels can be increased by USB interfacing with a microcontroller and by clustering more than one Raspberry Pi.

However, the high processing power of Raspberry Pi (28 nm technology), is accompanied by increased heat generation. This is not a major limitation, even when used for an extended, as ventilation from a heat sink or a running fan can attend to this issue. Without an external power supply, a single 8.4 V rechargeable lithium battery can power the system for about 10 days despite containing some unused peripherals on the STM32F407 development board. The need to integrate an autonomous power supply, such as a solar panel or other related technology, should be considered in future studies. Apparent limitations of the SPAC curve results include the relatively small sample of ambient noise data (collected within a time frame of about eight hours), short geophone spacing, and a short traverse of 8 m [30,31,32,33,34,35,36].

Finally, the proposed seismograph has good potential for seismic big data acquisition and processing using Raspberry Pi. The processing intelligence of the proposed seismograph can be extended to high-performance computing applications, such as AI related to seismology. This is an eye opener for future studies.

## Data Availability

Not applicable.
